# Association between postoperative nasogastric tube retention duration and total postoperative opioid consumption in patients undergoing minimally invasive esophagectomy: A single-center retrospective cohort study

**DOI:** 10.1097/MD.0000000000050798

**Published:** 2026-09-25

**Authors:** Pengfei Chen, Bindong Xu, Hao Chen, Qiuxia Liu, Zongxiang Lin

**Affiliations:** a Department of Thoracic and Cardiovascular Surgery, Affiliated Hospital of Putian University, Putian City, Fujian Province, China.

**Keywords:** minimally invasive esophagectomy, nasogastric tube retention duration, postoperative opioid consumption, retrospective cohort study

## Abstract

Minimally invasive esophagectomy is the preferred treatment for esophageal cancer. However, postoperative opioid analgesics are commonly used, and a nasogastric tube (NGT) is usually retained after surgery. There are few studies on the correlation between postoperative NGT retention duration and total postoperative opioid consumption. This study investigates the correlation between postoperative opioid consumption and NGT retention duration in patients undergoing minimally invasive esophagectomy. This single-center retrospective cohort study collected data from patients with esophageal cancer who underwent minimally invasive esophagectomy at the Department of Cardiothoracic Surgery, Affiliated Hospital of Putian University from January 2020 to December 2024. A total of 168 patients were included and divided into groups based on whether the NGT retention duration exceeded 7 days. The total postoperative opioid consumption was calculated and converted to daily oral morphine equivalent doses. Univariate analysis showed that postoperative hospital stay, NGT retention duration, fasting days, ICU stay, and postoperative complication grade were significantly correlated with postoperative morphine dose. Multivariate linear regression analysis showed that compared with the group with NGT retention duration ≤ 7 days, the group with NGT retention duration > 7 days had an average increase of 77.13 mg in postoperative morphine consumption (95% confidence interval: 31.62–122.65, *P* = .001). After adjusting for all covariates, the β value and 95% CI were 52.71 (3.84–101.59). Subgroup analysis and curve fitting results showed a significant linear positive correlation between NGT retention duration and total postoperative morphine consumption (*P* for overall = .001). There is a significant linear positive correlation between postoperative opioid consumption and NGT retention duration in patients undergoing minimally invasive esophagectomy. Clinicians should pay attention to the impact of NGT retention duration on postoperative pain. By optimizing postoperative management strategies and reducing unnecessary NGT retention duration, the quality of postoperative recovery can be improved.

## 1. Introduction

According to the 2022 Global Cancer Statistics Report, esophageal cancer ranks 11th and seventh in terms of incidence and mortality among global malignant tumors, respectively. In China, both the incidence and mortality of esophageal cancer are the highest in the world.^[1]^ Minimally invasive esophagectomy is the preferred treatment for esophageal cancer. Thoracic surgery is a more challenging field compared with other surgeries, with higher incidences of pain and pulmonary complications. Sufficient postoperative analgesia is crucial for surgical outcomes. Postoperative analgesia is usually achieved through the use of opioid analgesics, which are well-tolerated by patients and can maintain hemodynamic stability during the perioperative period.^[2]^ However, the use of opioids during the perioperative period is not without risks. Common complications include respiratory depression, postoperative nausea and vomiting, opioid-induced hyperalgesia, constipation, urinary retention, immune dysregulation, and neurotoxicity.^[3]^ Moreover, postoperative opioid use seems to trigger the development of opioid addiction, leading to the opioid abuse epidemic worldwide.^[4]^ Several studies have found correlations between postoperative opioid use, chronic pain development, and opioid addiction.^[5]^ Experimental and clinical studies have shown that different intraoperative anesthesia regimens or analgesics, including opioids, may affect oncological survival.^[6]^ Currently, there are 2 retrospective studies on the impact of perioperative opioid use and μ-opioid receptor expression on esophageal squamous cell carcinoma. The results indicate that high opioid use and increased μ-opioid receptor expression are associated with shorter survival. Compared with low-dose opioid use, high-dose opioid use during and after surgery is significantly related to increased recurrence risk.^[7,8]^ Therefore, analyzing the factors affecting opioid use after esophagectomy and reducing opioid use is clinically significant.

The traditional view is that NGT retention after esophagectomy can reduce anastomotic tension and lower the incidence of pulmonary infection,^[9]^ which is a common practice in clinical settings. In recent years, an increasing number of studies have shown that prolonged NGT retention after esophagectomy can cause complications such as nasal mucosal injury, epistaxis, sinusitis, dysphagia, dry throat, sore throat, severe nausea, vomiting, anastomotic tissue congestion and edema, and electrolyte disturbances.^[10-12]^ It does not reduce the incidence of anastomotic leakage and pulmonary infections. Early removal of NGT can reduce the fear and discomfort it causes to patients, help reduce postoperative opioid use, improve the quality of postoperative recovery, and even improve long-term survival.^[13]^ To our knowledge, there are few studies on the correlation between NGT retention duration and total opioid consumption after minimally invasive esophagectomy in esophageal cancer patients. Therefore, we conducted a retrospective cohort study to analyze the relationship between NGT retention duration and total opioid consumption after minimally invasive esophagectomy in esophageal cancer patients.

## 2. Materials and methods

### 2.1. Study design and participants

This single-center retrospective cohort study was conducted at the Affiliated Hospital of Putian University. Data were collected from patients with esophageal cancer who underwent radical esophagectomy at the Department of Cardiothoracic Surgery of the Affiliated Hospital of Putian University from January 2020 to December 2024. The inclusion criteria were as follows: Pathologically confirmed esophageal squamous cell carcinoma by biopsy under an electronic gastroscope; clinical stage T1-3N0-2M0 determined by preoperative examinations including enhanced CT of the neck, chest, and upper abdomen, and ultrasonography of the neck lymph nodes (according to the 10th edition of the American Joint Committee on Cancer [AJCC] cancer staging in 2009); age between 20 and 80 years; no severe abnormalities in cardiac, pulmonary, hepatic, or renal function; no contraindications to surgery; underwent minimally invasive esophagectomy (McKeown procedure); and complete clinical data. The exclusion criteria were as follows: Patients with cervical esophageal cancer (patients with non-squamous cell carcinoma in postoperative pathology); patients who underwent open surgery; patients who underwent intrathoracic anastomosis; patients who underwent esophageal replacement with colon or jejunum; and patients with contraindications to surgery or unable to tolerate surgery. A total of 178 patients were initially included. Two patients with non-squamous cell carcinoma in postoperative pathology, 4 patients who underwent open surgery, 2 patients who underwent intrathoracic anastomosis, and 2 patients who underwent esophageal replacement with colon or jejunum were excluded. Finally, 168 patients were included, comprising 114 males and 54 females. According to the clinical practice of this center and other major centers,^[14]^ NGT is routinely retained for 7 days after surgery before considering removal. Therefore, patients were divided into groups based on whether the NGT retention duration exceeded 7 days. The flowchart of patient selection is shown in Figure 1.

**Figure 1.**
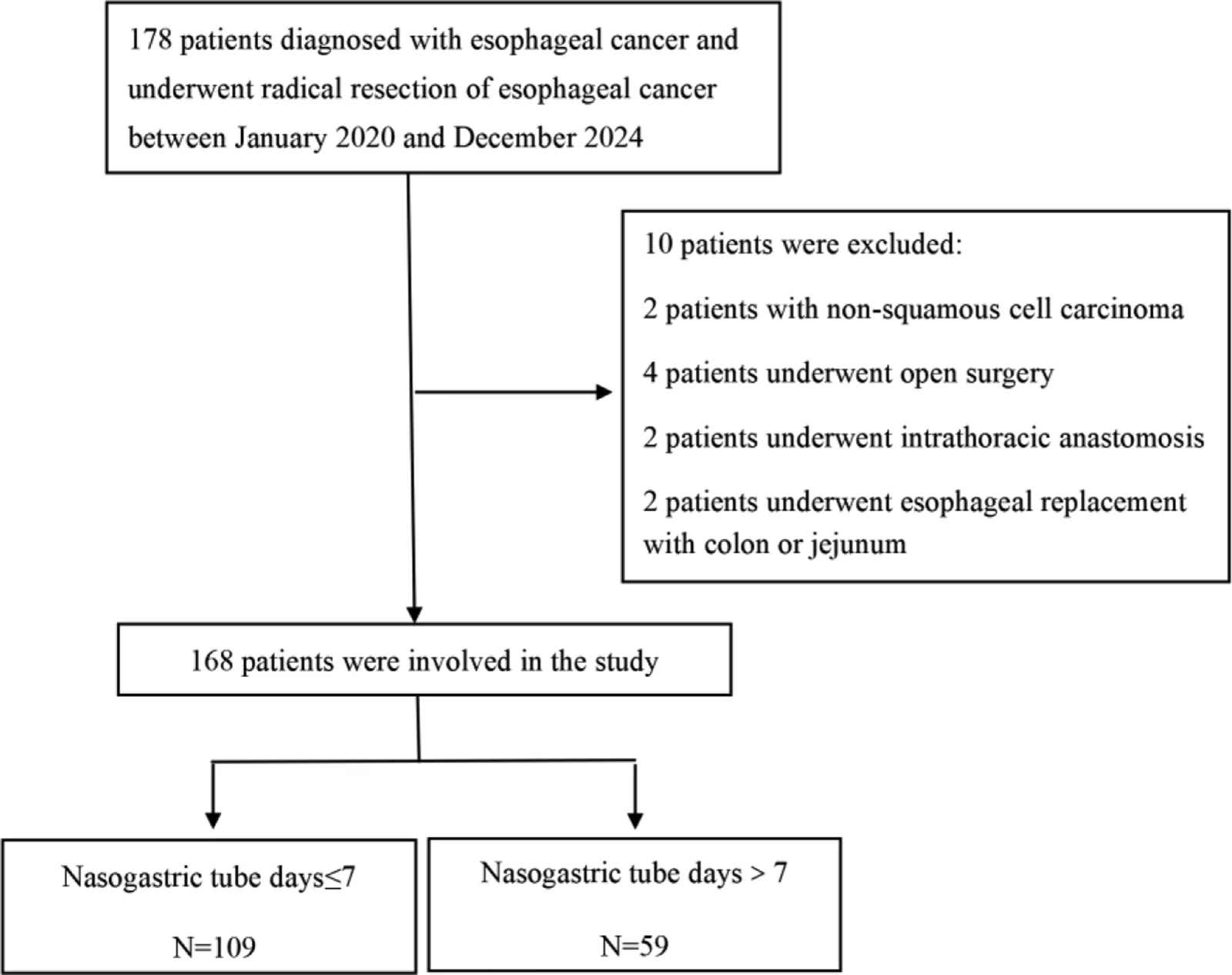
Flowchart of patient selection. A total of 178 patients with esophageal cancer who underwent radical resection between January 2020 and December 2024 were initially assessed. Ten patients were excluded: 2 with non-squamous cell carcinoma, 4 who underwent open surgery, 2 who underwent intrathoracic anastomosis, and 2 who underwent esophageal replacement with colon or jejunum. The remaining 168 patients were divided into 2 groups according to postoperative NGT retention duration: ≤7 days (n = 109) and >7 days (n = 59). NGT = nasogastric tube.

### 2.2. Ethics

This study adhered to the principles of the Helsinki Declaration and was approved by the Ethics Review Committee of the Affiliated Hospital of Putian University (Ethics approval number: 2025225). Considering the use of anonymized patient data, the requirement for informed consent was waived.

### 2.3. Surgical procedure

All patients underwent minimally invasive esophagectomy (McKeown procedure). Tumor margins were resected with at least 6 to 8 cm of normal tissue to avoid tumor involvement of the resection margins. Routine 2-field lymph node dissection was performed, with 3-field dissection if necessary. For reconstruction, the stomach was used as the substitute in all patients, with the anastomosis located in the left neck. an NGT was placed at the distal end of the residual stomach for adequate drainage.

### 2.4. Anesthesia

General anesthesia combined with epidural anesthesia was used, with single-lumen endotracheal intubation and right lung isolation.

### 2.5. Postoperative management

All patients were routinely fasted after surgery. Within 24 hours postoperatively, 500 ml of enteral nutrition solution was administered through a duodenal nutrition tube, with a gradual increase in dosage. A nutritionist was consulted to provide combined intravenous and enteral nutrition support based on the patient’s physiological needs and biochemical indicators. Prophylactic antibiotics, proton pump inhibitors, and gastrointestinal motility-promoting drugs were administered. Adequate analgesia was ensured, and patients were encouraged to mobilize early or stand by the bedside. After confirming no anastomotic leakage by drinking 50 mL of methylene blue, oral intake of a liquid diet was initiated, gradually transitioning to a semi-liquid diet.

### 2.6. Nasogastric tube retention duration

A NGT was routinely placed postoperatively and continuously drained. The NGT retention duration was calculated based on the appearance and volume of the drainage fluid, as well as x-ray confirmation of no gastric emptying disorder or gastric dilation.

### 2.7. Opioid consumption statistics and dose conversion

The names, dosages, and total amounts of various opioid analgesics used by patients in the postoperative period, including patient-controlled analgesia (PCA), intensive care unit, and ward, were recorded and converted to daily oral morphine equivalent doses using the conversion formula shown in Table 1.

**Table 1:** Conversion of opioid analgesic doses.

Category	Drug name	Original dose (mg)	Approximate oral morphine equivalent dose (mg)
Weak opioids	Codeine tablets	1	2/13
	Codeine oral solution	1	2/13
	Oxycodone hydrochloride extended-release tablets	1	2
	Morphine sulfate extended-release tablets	1	1
	Sufentanil injection	1 μg	1
	Remifentanil injection	1 μg	0.1
	Alfentanil	1	10–15
Strong opioids	Morphine hydrochloride injection	1	3
	Hydromorphone hydrochloride injection	1	5
	Buprenorphine injection	1	5
	Dizocilpine injection	1	3
	Pentazocine injection	3	1
	Buprenorphine injection	1	1
Combination products	Lofentanil controlled-release tablets (contains codeine 13 mg)	1 tablet	2

### 2.8. Covariates

Baseline demographic, surgical, and postoperative data were obtained from patient interviews and medical records.

The following covariates were analyzed based on previous studies^[15,16]^ and clinical significance: Epidemiological and preoperative clinical characteristics, such as age, gender, body mass index (BMI), education level, smoking history, alcohol history, previous surgical history, preoperative neoadjuvant treatment, Charlson comorbidity score,^[17]^ and American Society of Anesthesiologists physical status classification^[18]^; surgical factors: surgical time, intraoperative blood loss, number of lymph nodes dissected, and number of postoperative drainage tubes; postoperative factors: postoperative pathological stage, postoperative complications graded according to the Clavien-Dindo classification,^[19]^ postoperative ICU stay, and postoperative hospital stay.

### 2.9. Data definitions

BMI (kg/m^2^) was calculated by dividing weight (kg) by the square of height (m). According to Chinese adult weight standards, weight was categorized into 4 classes based on BMI: underweight (<18.5 kg/m^2^), normal weight (18.5–23.9 kg/m^2^), overweight (24.0–27.9 kg/m^2^), and obese (≥28.0 kg/m^2^).^[20]^ Preoperative neoadjuvant treatment refers to a series of treatments implemented before surgery in patients with resectable locally advanced esophageal squamous cell carcinoma. The aim is to shrink the tumor volume, reduce the tumor stage, and thereby increase the surgical resection rate, reduce the risk of postoperative recurrence and metastasis, and improve long-term survival outcomes. In this study, neoadjuvant treatments included chemotherapy, radiotherapy, and immunotherapy.^[21]^ The Charlson Comorbidity Index (CCI) is a quantitative tool used to assess the severity of patients’ comorbidities. It predicts long-term mortality risk and disease burden by considering various chronic diseases and their severity. The index includes 19 common chronic diseases, each assigned a weight score ranging from 1 to 6 based on its impact on patient prognosis. The total score, obtained by summing the weight scores of all comorbidities, indicates the severity of comorbidities and long-term mortality risk.^[17]^ The American Society of Anesthesiologists Physical Status Classification System (ASA grading) is used to assess patients’ preoperative physical condition. It categorizes patients into 6 grades, from 1 (healthy) to 6 (brain dead, for organ transplantation), with the option to add “E” for emergency surgery.^[18]^ Surgical time is measured from the start of the first surgical skin incision to the completion of the last skin suture. Intraoperative blood loss is recorded by the surgeon. The number of postoperative drainage tubes is the total count of drainage tubes left in place in the neck, chest, mediastinum, and abdomen. Postoperative complications are defined according to the first Chinese Terminology Collection of Thoracic Surgery. Complication grading follows the internationally recognized Clavien-Dindo classification.^[19]^ The Clavien-Dindo classification is a system for assessing the severity of postoperative complications. It categorizes complications into 5 grades: Grade I for mild complications requiring pharmacological treatment; grade II for complications requiring interventional treatment but not affecting daily activities; grade III for complications requiring surgical, endoscopic, or radiological interventional treatment, further divided into IIIa (non-life-threatening) and IIIb (requiring ICU treatment); grade IV for life-threatening complications, divided into IVa (single-organ failure) and IVb (multi-organ failure); grade V for death.

### 2.10. Statistical analysis

This study aimed to investigate the impact of postoperative NGT retention duration on total postoperative opioid consumption in patients undergoing minimally invasive esophagectomy. Patients were divided into 2 groups based on whether the NGT retention duration exceeded 7 days. A descriptive analysis was performed for all participants.

All normally distributed and skewed continuous variables are expressed as mean (standard deviation) or median (interquartile range). Categorical variables are presented as frequency (%).

Student *t* test or Mann–Whitney *U*-test was used to compare continuous variables between groups based on the normality of distribution. Fisher exact test or χ^2^ test was employed to compare categorical variables between groups.

Linear regression models were used to study the association between postoperative NGT retention duration and total postoperative opioid consumption in patients undergoing minimally invasive esophagectomy, controlling for the effects of age, gender, body mass index (BMI), education level, smoking history, alcohol history, previous surgical history, preoperative neoadjuvant treatment, Charlson comorbidity score, American Society of Anesthesiologists physical status classification, surgical time, intraoperative blood loss, number of lymph nodes dissected, number of postoperative drainage tubes, postoperative pathological stage, postoperative complications graded according to the Clavien-Dindo classification, postoperative ICU stay, and postoperative hospital stay.

Unadjusted and multivariate models were used. The multivariate model included factors of clinical concern and covariates reported in previous studies. Other potential confounding factors were selected based on previous scientific data or a change of >10% in effect estimates. Three models were created: Model 1 adjusted for age and gender; model 2 further adjusted for smoking history, previous surgical history, and preoperative neoadjuvant treatment; model 3 also adjusted for Charlson comorbidity score, surgical time, intraoperative blood loss, number of lymph nodes dissected, number of postoperative drainage tubes, postoperative pathological stage, postoperative complications graded according to the Clavien-Dindo classification, and postoperative ICU stay (Table 3).

*P*-values < .05 were considered statistically significant. All analyses were performed using the statistical software package R 3.3.2 (http://www.R-project.org, The R Foundation) and Free Statistics analysis platform (Version 1.1).

## 3. Results

### 3.1. Baseline characteristics of study participants according to nasogastric tube retention duration

A total of 178 patients with esophageal cancer who underwent minimally invasive esophagectomy at the Department of Cardiothoracic Surgery, Affiliated Hospital of Putian University, from January 2020 to December 2024 were initially included, with 169 patients meeting the criteria for analysis (Fig. 1). Table 2 reports the descriptive characteristics of the study population, divided into groups based on NGT retention duration: ≤7 days and >7 days. There were no statistically significant differences between the 2 groups in demographic characteristics such as age, gender, and BMI. Lifestyle factors such as education level, smoking history, and alcohol history also showed no significant differences between the groups. In terms of preoperative and surgical characteristics, the 2 groups had similar distributions of preoperative neoadjuvant treatment (*P* = .514), previous surgical history (*P* = .509), ASA grading (*P* = .52), and CCI score (*P* = .581). Surgical time (*Q*1: 299.6 ± 63.6 minutes, *Q*2: 326.6 ± 52.5 minutes, *P* = .006) and intraoperative blood loss (*Q*1: 176.1 ± 37.6 mL, *Q*2: 191.5 ± 57.3 mL, *P* = .038) were significantly different between the 2 groups, while the number of lymph nodes dissected (*P* = .105) and postoperative pathological stage (*P* = .951) were not significantly different. Postoperative characteristics revealed that *Q*2 had a significantly longer postoperative hospital stay (23.3 ± 8.9 days) compared with *Q*1 (14.8 ± 4.9 days, *P* < .001), a longer NGT retention duration (median 11.0 days) compared with *Q*1 (median 6.0 days, *P* < .001), and more fasting days (17.9 ± 8.5 days) compared with *Q*1 (10.8 ± 4.5 days, *P* < .001). Postoperative complication grade (*P* = .004) and ICU stay (*Q*1: 0.4 ± 1.4 days, *Q*2: 1.0 ± 2.2 days, *P* = .041) also showed significant differences between the 2 groups. In terms of total postoperative opioid consumption, the median postoperative morphine dose in *Q*2 was 321.5 mg, significantly higher than 262.0 mg in *Q*1 (*P* = .002). Overall, patients with longer NGT retention duration exhibited longer hospital stays, fasting periods, and higher opioid consumption during postoperative recovery.

**Table 2:** Baseline patient characteristics according to nasogastric tube days in esophageal cancer patients.

Variables	Total	Nasogastric tube days		*P* value
		*Q*1 (≤7 d)	*Q*2 (>7 d)	
Participants, n	168	109	59	
Age (yr)	65.0 ± 8.2	64.6 ± 8.6	65.8 ± 7.3	.345
Sex, n (%)				.481
Male	114 (67.9)	76 (69.7)	38 (64.4)	
Female	54 (32.1)	33 (30.3)	21 (35.6)	
BMI	22.0 ± 3.1	22.1 ± 3.2	21.9 ± 3.0	.671
Education, n (%)				.845
Junior high school or less	138 (82.1)	90 (82.6)	48 (81.4)	
High school or above	30 (17.9)	19 (17.4)	11 (18.6)	
Smoking history, n (%)				.235
No	149 (88.7)	99 (90.8)	50 (84.7)	
Yes	19 (11.3)	10 (9.2)	9 (15.3)	
Drinking history, n (%)				.658
No	163 (97.0)	105 (96.3)	58 (98.3)	
Yes	5 (3.0)	4 (3.7)	1 (1.7)	
Preoperative neoadjuvant therapy history, n (%)				.514
No	141 (83.9)	90 (82.6)	51 (86.4)	
Yes	27 (16.1)	19 (17.4)	8 (13.6)	
Previous surgical history, n (%)				.509
No	144 (85.7)	92 (84.4)	52 (88.1)	
Yes	24 (14.3)	17 (15.6)	7 (11.9)	
ASA classification, n (%)				.52
Grade I	157 (93.5)	103 (94.5)	54 (91.5)	
Grade II	11 (6.5)	6 (5.5)	5 (8.5)	
CCI score, n (%)				.581
1	36 (21.4)	26 (23.9)	10 (16.9)	
2	113 (67.3)	71 (65.1)	42 (71.2)	
3	19 (11.3)	12 (11)	7 (11.9)	
Operating time (min)	309.1 ± 61.2	299.6 ± 63.6	326.6 ± 52.5	.006
Bleeding volume (mL)	181.5 ± 46.0	176.1 ± 37.6	191.5 ± 57.3	.038
Number of lymph nodes dissected	27.3 ± 10.9	28.3 ± 11.3	25.5 ± 9.8	.105
Pathologic stage, n (%)				.951
Ia	28 (16.7)	18 (16.5)	10 (16.9)	
Ib	23 (13.7)	14 (12.8)	9 (15.3)	
IIa	15 (8.9)	10 (9.2)	5 (8.5)	
IIb	42 (25.0)	29 (26.6)	13 (22)	
IIIa	11 (6.5)	6 (5.5)	5 (8.5)	
IIIb	40 (23.8)	27 (24.8)	13 (22)	
IVa	9 (5.4)	5 (4.6)	4 (6.8)	
Postoperative stay	17.8 ± 7.7	14.8 ± 4.9	23.3 ± 8.9	<.001
Number of postoperative drains, n (%)				<.001
3	2 (1.2)	0 (0)	2 (3.4)	
4	77 (45.8)	61 (56)	16 (27.1)	
5	61 (36.3)	39 (35.8)	22 (37.3)	
6	24 (14.3)	9 (8.3)	15 (25.4)	
7	3 (1.8)	0 (0)	3 (5.1)	
8	1 (0.6)	0 (0)	1 (1.7)	
Postoperative fasting days	13.3 ± 7.0	10.8 ± 4.5	17.9 ± 8.5	<.001
ICU stay	0.6 ± 1.7	0.4 ± 1.4	1.0 ± 2.2	.041
Postoperative complication grade, n (%)				.004
<Grade II	117 (69.6)	84 (77.1)	33 (55.9)	
≥Grade II	51 (30.4)	25 (22.9)	26 (44.1)	
Postoperative morphine dose	271.5 (224.0, 334.2)	262.0 (216.5, 307.0)	321.5 (243.0, 411.0)	0.002

Data presented are mean ± SD, median (*Q*1–*Q*3), or N (%).

ASA = The American Society of Anesthesiologists, BMI = body mass index, CCI = Charlson Comorbidity Index, ICU = intensive care unit, SD = standard deviation.

### 3.2. Univariate analysis of factors associated with postoperative opioid consumption

Table 3 presents the results of univariate analysis of factors associated with total postoperative opioid consumption in patients undergoing minimally invasive esophagectomy, reported as β values and 95% confidence intervals. Univariate analysis showed that postoperative hospital stay, NGT retention duration, fasting days, ICU stay, and postoperative complication grade (Clavien-Dindo Grade 1) were significantly correlated with postoperative morphine dose. In contrast, age, BMI, education level, smoking history, alcohol history, preoperative neoadjuvant treatment, previous surgical history, ASA grading, CCI score, and postoperative pathological stage were not significantly correlated with postoperative morphine dose.

**Table 3:** Univariate analysis of factors associated with total postoperative opioid consumption in patients undergoing minimally invasive esophagectomy.

Variables	β (95% CI)	*P (t* test)	*P (F*-test)
Age (cont. var.)	0.84 (−1.93, 3.61)	.55	.55
Sex			
Man	Ref.		
Female	−70.7 (−117.86, −23.54)	.004	.004
BMI (cont. var.)	0.9 (−6.43, 8.24)	.808	.808
Education			
Junior high school or less	Ref.		
High school or above	11.4 (−47.58, 70.38)	.703	.703
Smoking history			
No	Ref.		
Yes	32.74 (−38.44, 103.92)	.365	.365
Drinking history			
No	Ref.		
Yes	−52.4 (−185.15, 80.36)	.437	.437
Preoperative neoadjuvant therapy history			
No	Ref.		
Yes	18.03 (−43.44, 79.5)	.563	.563
Previous surgical history			
No	Ref.		
Yes	30.72 (−33.69, 95.14)	.348	.348
ASA classification			
0	Ref.		
1	−32.52 (−123.75, 58.71)	.483	.483
CCI score			.781
1	Ref.		
2	15.31 (−40.83, 71.46)	.591	
3	27.98 (−55.22, 111.17)	0.508	
Operating time (cont. var.)	0.53 (0.17, 0.89)	.004	.004
Bleeding volume (cont. var.)	0.26 (−0.23, 0.75)	.3	.3
Number of lymph nodes dissected (cont. var.)	1.11 (−0.97, 3.18)	.294	.294
Pathologic stage			.607
Ia	Ref.		
Ib	52.41 (−30.16, 134.98)	.212	
IIa	30.87 (−63.02, 124.75)	.517	
IIb	27.02 (−44.57, 98.6)	.457	
IIIa	7.87 (−96.54, 112.28)	.882	
IIIb	16.02 (−56.28, 88.32)	.662	
IVa	105.57 (−6.86, 218)	.066	
Postoperative stay (cont. var.)	6.63 (3.88, 9.38)	<.001	<.001
Number of postoperative drains			<.001
3	Ref.		
4	−75.23 (−274.18, 123.72)	.456	
5	−71.59 (−271.2, 128.02)	.48	
6	−28.29 (−232.73, 176.14)	.785	
7	41.17 (−212.4, 294.74)	.749	
8	540.5 (200.3, 880.7)	.002	
Nasogastric tube days (cont. var.)	8.03 (4.49, 11.58)	<.001	<.001
Postoperative fasting days (cont. var.)	5.35 (2.24, 8.46)	<.001	<.001
ICU stay (cont. var.)	26.71 (14.28, 39.15)	<.001	<.001
Postoperative complication grade			
0	Ref.		
1	77.78 (30.1, 125.47)	.002	.002

ASA = The American Society of Anesthesiologists, BMI = body mass index, CCI = Charlson Comorbidity Index, ICU = intensive care unit.

### 3.3. Association between postoperative NGT retention duration and postoperative morphine consumption

The results of multivariate linear regression analysis on the association between postoperative NGT retention duration and postoperative morphine consumption in patients undergoing minimally invasive esophagectomy are shown in Table 4.

**Table 4:** Multivariate regression analysis.

Variable	Non‑adjusted model		Model I		Model II		Model III	
	β (95% CI)	*P*-value	β (95% CI)	*P*-value	β (95% CI)	*P*-value	β (95% CI)	*P*-value
Nasogastric tube days	8.03 (4.52–11.55)	<.001	7.72 (4.28–11.17)	<.001	7.86 (4.35–11.37)	<.001	7.66 (3.9–11.41)	<.001
Binary variable								
≤7 d	Ref.		Ref.		Ref.		Ref.	
>7 d	77.13 (31.62–122.65)	0.001	79.66 (35.25–124.06)	0.001	81.43 (36.39–126.47)	0.001	52.71 (3.84–101.59)	.036

Model I: Adjusted for Age + Sex; model II: Adjusted for model I + Smoking history + Preoperative neoadjuvant therapy history + Previous surgical history; model III: Adjusted for model II + CCI score + Operating time + Bleeding volume + Number of lymph nodes dissected + Number of postoperative drains + Pathologic stage + Postoperative complication grade + ICU stay.

ASA = The American Society of Anesthesiologists, BMI = body mass index, CCI = Charlson Comorbidity Index, CI = confidence interval, ICU = intensive care unit, ref = reference group, β = beta.

Table 4 summarizes the β values and corresponding CIs for postoperative morphine consumption based on NGT retention duration and whether the NGT retention duration exceeded 7 days. Compared with the group with NGT retention duration ≤7 days, the group with NGT retention duration >7 days had an average increase of 77.13 mg in postoperative morphine consumption (95% confidence interval: 31.62–122.65, *P* = .001). After adjusting for all covariates, the β value and 95% CI were 52.71 (3.84–101.59). The statistical results of all models were robust (Table 4).

### 3.4. Subgroup analysis

Further subgroup analysis was conducted in this study to explore the impact of different patient characteristics on the association between postoperative NGT retention duration and total postoperative opioid consumption. The subgroup analysis showed that there was a significant positive correlation between NGT retention duration and postoperative morphine consumption in patients with different genders, ages, CCI scores, BMIs, smoking histories, preoperative neoadjuvant treatment histories, previous surgical histories, postoperative pathological stages, and postoperative complication grades (Fig. 2). However, factors such as gender, age, CCI score, BMI, smoking history, preoperative neoadjuvant treatment history, and previous surgical history did not significantly affect this association (all interaction *P*-values > .05). This indicates that the impact of NGT retention duration on total postoperative opioid consumption was consistent across different subgroups.

**Figure 2.**
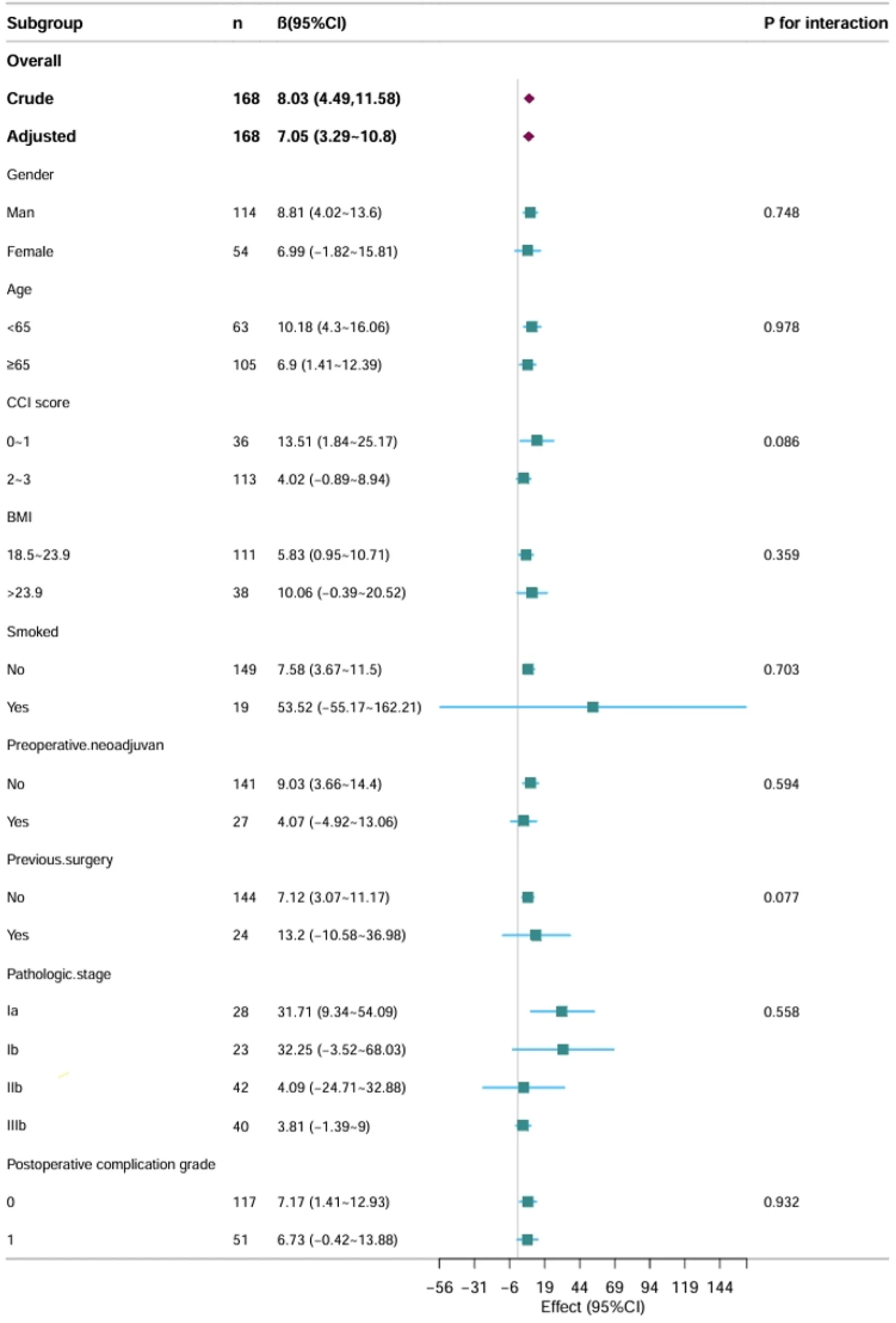
Subgroup analysis of the association between nasogastric tube retention duration and postoperative morphine consumption. Subgroup analyses were stratified by gender, age, CCI score, BMI, smoking history, preoperative neoadjuvant therapy history, previous surgical history, pathologic stage, and postoperative complication grade. Each stratification was adjusted for age, sex, smoking history, preoperative neoadjuvant therapy history, previous surgical history, CCI score, operating time, bleeding volume, number of lymph nodes dissected, number of postoperative drains, pathologic stage, postoperative complication grade, and ICU stay, except for the stratification factor itself. The effect estimates represent the increase in postoperative morphine dose (mg) per additional day of NGT retention. *P* values for interaction were all >.05, indicating no significant effect modification by any of the subgroup variables. BMI = body mass index, CCI = Charlson Comorbidity Index, CI = confidence interval, β = beta coefficient.

### 3.5. Curve fitting

There was a significant linear positive correlation between NGT retention duration and postoperative morphine consumption (*P* for overall = .001; Fig. 3). With each additional day of NGT retention, the total postoperative morphine consumption significantly increased.

**Figure 3.**
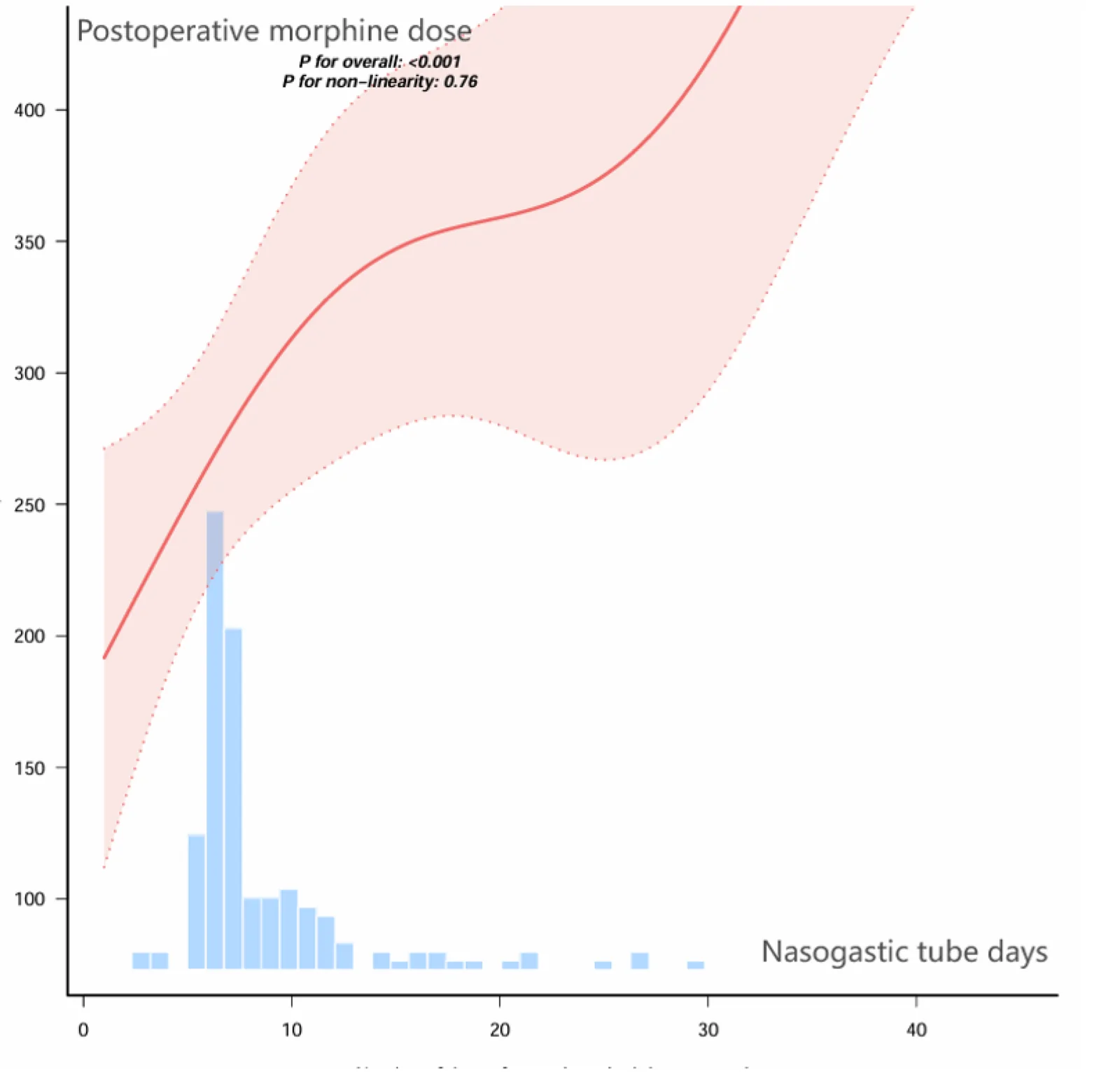
Dose–response relationship between nasogastric tube retention duration and postoperative morphine consumption. Smoothing splines were generated by generalized additive models. The solid line represents the fitted curve, and the shaded area represents the 95% confidence interval. Adjusted for age, sex, smoking history, preoperative neoadjuvant therapy history, previous surgical history, CCI score, operating time, bleeding volume, number of lymph nodes dissected, number of postoperative drains, pathologic stage, postoperative complication grade, and ICU stay. CI = confidence interval, ICU = intensive care unit.

## 4. Discussion

In this retrospective study of patients with esophageal cancer who underwent minimally invasive esophagectomy, we investigated the relationship between postoperative opioid consumption and NGT retention duration through univariate analysis, multivariate analysis, and subgroup analysis. The results of univariate analysis showed that male gender, surgical time, NGT retention duration, number of postoperative drainage tubes, fasting days, ICU stay, postoperative hospital stay, and postoperative complication grade (Clavien-Dindo grade) were significantly correlated with postoperative morphine dose. In contrast, age, BMI, education level, smoking history, alcohol history, preoperative neoadjuvant treatment, previous surgical history, preoperative ASA grading, preoperative CCI score, and postoperative pathological stage were not significantly correlated with postoperative morphine dose. Curve fitting results showed a significant linear positive correlation between NGT retention duration and total postoperative morphine consumption (*P* for overall = .001). This indicates that with the prolongation of NGT retention duration, the total postoperative morphine consumption significantly increased. This relationship was robust in both unadjusted and stepwise adjusted analyses and remained consistent across different subgroups. This finding suggests that NGT retention duration may be an important factor affecting total postoperative opioid consumption.

Reviewing the literature, Franqueiro et al reported that females are more likely to experience postoperative pain after thoracic surgery.^[22]^ Fan et al indicated that surgical time is a risk factor for postoperative pain in esophageal cancer.^[23]^ Sun et al found that postoperative pain after thoracoscopic surgery is related to the number of drainage tubes.^[24]^ Tong et al reported that gender, age, surgical time, drainage tube status, chemotherapy history, and education level are predictors of postoperative pain.^[25]^ Kinney et al found that a prolonged hospital stay after chest surgery increases the occurrence of postoperative pain and opioid use.^[26]^ Hoelzen et al reported that groups with lower postoperative complication rates, shorter hospital stays, and shorter postoperative ICU stays had lower total postoperative opioid use.^[27]^ Tak Kyu Oh et al reported that chronic smoking is not associated with increased postoperative opioid use in patients with lung cancer or esophageal cancer.^[28]^ These study results are similar to our univariate analysis results, except for gender. Our study found that female patients had lower total postoperative opioid consumption, which may be related to the higher fat content in the female chest wall, resulting in better tolerance to chest drainage tubes. It may also be related to the fact that most women have experienced the severe pain of childbirth, which has increased their pain threshold. Of course, some scholars have results similar to ours. For example, Bergljot R Karlsdottir et al found in a retrospective study of 592 patients with colon cancer who underwent elective colorectal resection that male gender, smoking, younger age, and preoperative pain increased postoperative opioid demand.^[16]^

Esophageal cancer is one of the most common cancers worldwide, with over 6,00,000 new cases in 2020. Despite advancements in perioperative oncological treatment that have improved survival rates, surgical resection remains the primary treatment for esophageal cancer and gastroesophageal junction cancer. However, this surgery is associated with a high rate of complications that may impact long-term survival and cancer recurrence. Therefore, standardized surgical procedures and perioperative management protocols have been widely adopted to prevent and reduce the potential consequences of complications. Nevertheless, there are differences in perioperative treatment strategies and protocols among different institutions, and many guidelines are based on limited evidence. Particularly, there is heterogeneity in the use of gastric pyloric drainage, nutritional jejunostomy, and nasogastric tube (NGT) placement. NGT placement is considered one of the most uncomfortable aspects of postoperative care by many patients and may interfere with the implementation of enhanced recovery after surgery (ERAS) protocols.^[29]^ NGTs are routinely used in patients undergoing gastrointestinal surgery to prevent abdominal distension, reduce the risk of aspiration and pneumonia, promote early recovery of gastrointestinal function, and facilitate early discharge. The traditional view is that NGT retention after esophagectomy can reduce anastomotic tension and lower the incidence of pulmonary infection.^[9]^ However, prolonged NGT retention can cause significant discomfort to patients, thereby increasing postoperative opioid use. The incidence of serious complications and mortality after esophagectomy is high, and there is still controversy regarding early NGT removal.^[30]^ Those who support routine NGT placement believe that since the reconstructed stomach and anastomosis are located proximal to the NGT tip, NGTs may be more effective in patients with esophagectomy.^[31]^ Enhanced recovery after surgery (ERAS) refers to a series of optimized measures for perioperative management to reduce physical and psychological trauma stress in surgical patients, thereby accelerating patient recovery. The use of minimally invasive surgery, early NGT removal, and postoperative pain control are important cornerstones of ERAS programs.^[32]^ ERAS has been successfully implemented in the surgical treatment of esophageal cancer.^[33,34]^ ERAS for esophageal cancer emphasizes the avoidance of tubes and fasting.^[35]^ Masato Hayashi et al believe that although early NGT removal did not reduce the incidence of postoperative pneumonia, this practice is safe. Therefore, NGT removal can be performed earlier than the traditional 7 days.^[14]^ Vignesh et al also reached the same conclusion in their published paper. Conventional NGT placement after minimally invasive esophagectomy increases patient discomfort, delays mobilization and oral intake, and early NGT removal can reduce these discomforts, improve patient compliance with ERAS protocols, and is not associated with an increased risk of anastomotic leakage. It is also related to a shorter hospital stay.^[36]^

Our study showed that with the prolongation of NGT retention duration, total postoperative opioid consumption significantly increased and remained consistent across different subgroups. This may be related to the following factors:Gastrointestinal dysfunction and postoperative discomfort: The main function of NGT is to reduce the tension in the gastrointestinal tract by negative pressure suction, relieving gas and fluid accumulation and promoting postoperative gastrointestinal function recovery. However, prolonged NGT retention may inhibit the normal function of the gastrointestinal tract, resulting in weakened gastrointestinal motility and reduced digestive juice secretion. This can lead to symptoms such as abdominal distension, nausea, and vomiting.^[37]^ These discomforts increase patients’ pain perception, thereby requiring more opioids for pain relief. Moreover, long-term NGT retention may cause local mucosal injury or inflammatory response, further exacerbating patients’ discomfort and pain.^[12]^ Impact of postoperative complications: Longer NGT retention duration may be associated with the occurrence of postoperative complications. For example, prolonged NGT retention may increase the risk of infection, leading to infectious complications. Infectious complications not only cause systemic inflammatory responses but also lead to increased local pain, thereby increasing postoperative morphine consumption. In addition, prolonged NGT retention duration may be related to gastrointestinal dysfunction and anastomotic leakage, which also increase patients’ pain perception and demand for analgesics. Prolonged postoperative hospital stay: Our univariate analysis showed a significant positive correlation between postoperative hospital stay and postoperative morphine consumption (*P* < .001). This indicates that the longer the hospital stay, the longer the duration of postoperative opioid analgesia, resulting in increased total postoperative opioid consumption. Prolonged NGT retention duration may extend postoperative hospital stay, as doctors may need more time to observe and manage complications or discomforts related to NGT retention. Therefore, optimizing postoperative management strategies to reduce unnecessary hospital stay and NGT retention duration may help decrease postoperative opioid consumption, improve the quality of postoperative recovery, and enhance patient compliance with ERAS protocols, which is in line with the ERAS concept.^[36]^

The results of our study suggest that clinicians should pay attention to the impact of NGT retention duration on postoperative pain during postoperative management. Optimizing postoperative management strategies, such as early NGT removal, may help reduce postoperative pain and morphine consumption, thereby improving patients’ postoperative recovery experience. For example, for patients with good postoperative gastrointestinal function recovery, early NGT removal can be considered to reduce unnecessary retention duration. In addition, for patients with longer NGT retention duration, their pain conditions and complications should be closely monitored, and timely measures should be taken to alleviate their discomforts and reduce dependence on opioids, which may even improve long-term survival.^[13]^

Although our subgroup analysis showed that different patient characteristics (such as gender, age, CCI score, BMI, smoking history, etc) did not significantly affect the relationship between NGT retention duration and postoperative morphine consumption, this does not mean that these factors are unimportant in postoperative pain management. In fact, individualized pain management remains the key to postoperative analgesia. For example, for elderly patients or those with multiple underlying diseases, postoperative pain should be managed more cautiously to avoid adverse reactions caused by excessive morphine use. Moreover, for patients at high risk of postoperative complications, preventive and management plans should be formulated in advance to reduce the occurrence of complications, thereby decreasing postoperative pain and morphine consumption.

In addition to optimizing postoperative management strategies and individualized pain management, postoperative rehabilitation guidance is also of great significance for patient recovery. Healthcare professionals should provide detailed postoperative rehabilitation guidance to patients and their families, including dietary adjustments, activity guidance, and psychological support. For example, encouraging patients to mobilize early can promote gastrointestinal function recovery and reduce the occurrence of bloating and constipation. Moreover, reasonable dietary adjustments can also help alleviate the burden on the gastrointestinal tract and promote postoperative recovery. Through comprehensive postoperative rehabilitation guidance, the quality of postoperative recovery can be further improved, reducing postoperative pain and opioid consumption.

## 5. Study limitations

Several limitations of this study should be acknowledged. First, as a single-center retrospective cohort study, the findings may have limited generalizability to institutions with different perioperative protocols, pain management strategies, or NGT removal criteria. Second, due to the retrospective nature of the data, we were unable to capture patient-reported pain scores (e.g., visual analog scale or numeric rating scale) or quality-of-life measurements, which would have provided a more comprehensive assessment of the patients’ pain experience beyond opioid consumption alone. Third, although we adjusted for a broad range of covariates, we could not fully account for unmeasured confounders, including preoperative opioid use history, psychological distress (e.g., anxiety or depression), individual variations in pain perception, and differences in nursing care quality or postoperative pain assessment practices. These factors may introduce residual confounding and should be addressed in future prospective studies. Fourth, our study focused exclusively on in-hospital opioid consumption and did not evaluate long-term outcomes such as chronic post-surgical pain, opioid dependence, or survival, which are clinically important endpoints. Fifth, while we observed a significant association between prolonged NGT retention and increased opioid consumption, the underlying mechanisms remain speculative. Potential pathways – such as local mucosal irritation, pharyngeal and esophageal discomfort, inflammatory responses, or impaired mobilization due to tube-related distress – could not be directly examined in this retrospective design. Additionally, we did not perform a formal mediation analysis to quantify how specific postoperative complications (e.g., anastomotic leakage, pneumonia, or ileus) might mediate the relationship between NGT retention duration and pain intensity. Finally, the relatively modest sample size may limit the precision of our estimates and the generalizability of subgroup analyses. Therefore, larger multicenter prospective studies with standardized pain assessment tools and mechanistic evaluations are warranted to validate our findings and to explore the causal pathways underlying this association.

## 6. Conclusion

Through univariate and subgroup analyses, our study confirmed a significant linear positive correlation between NGT retention duration and postoperative morphine consumption. This finding suggests that clinicians should pay attention to the impact of NGT retention duration on postoperative pain during postoperative management. By optimizing postoperative management strategies to reduce unnecessary NGT retention duration, the quality of postoperative recovery can be improved. Future research could further explore the underlying mechanisms of this relationship and validate our results.

## Author contributions

**Conceptualization:** Hao Chen.

**Data curation:** Qiuxia Liu, Zongxiang Lin.

**Formal analysis:** Pengfei Chen, Bindong Xu.

**Funding acquisition:** Pengfei Chen.

**Investigation:** Pengfei Chen, Bindong Xu, Qiuxia Liu, Zongxiang Lin.

**Methodology:** Pengfei Chen, Hao Chen.

**Project administration:** Pengfei Chen.

**Resources:** Pengfei Chen.

**Software:** Bindong Xu.

**Supervision:** Hao Chen.

**Validation:** Pengfei Chen, Hao Chen.

**Visualization:** Pengfei Chen.

**Writing – original draft:** Pengfei Chen.

**Writing – review & editing:** Pengfei Chen, Bindong Xu, Hao Chen, Qiuxia Liu, Zongxiang Lin.
